# 6,9-Dimeth­oxy-3,4-dihydro-1*H*-1,4-oxazino[4,3-*a*]indol-1-one

**DOI:** 10.1107/S1600536811000249

**Published:** 2011-01-12

**Authors:** Cristian O. Salas, Ricardo A. Tapia, Yolanda Prieto

**Affiliations:** a Departamento de Química Orgánica, Facultad de Química, Pontificia Universidad Católica de Chile, 702843 Santiago de Chile, Chile

## Abstract

The title compound, C_13_H_13_NO_4_, is one cyclization product of the reaction of ethyl 1-(2-bromo­eth­yl)-4,7-dimeth­oxy-1*H*-indole-2-carboxyl­ate with sodium azide in refluxing dioxane and was synthesized with the aim of finding new compounds with biological properties. Bond lengths and angles are within the expected values and confirm the bond orders giving in the scheme. The shortest contacts between mol­ecules are set along the *a* axis, where stacked mol­ecules related by an inversion center form an *ABAB* array through π–π stacking inter­actions with centroid–centroid distances ranging from 3.922 (2) to 4.396 (2) Å. Weak C—H⋯O hydrogen bonds further stabilize the structure.

## Related literature

For background to oxazinoindoles as inter­mediates in the chemistry of bioactive compounds, see: Demerson *et al.* (1975 ▶); Fedouloff *et al.* (2001 ▶); Shchekotikhin *et al.* (2004 ▶). Several synthetic strategies for the preparation of oxazinoindoles have been reported, for some examples, see: Abbiati *et al.* (2005 ▶); Brudeli *et al.* (2010 ▶); Fu *et al.* (2010 ▶).
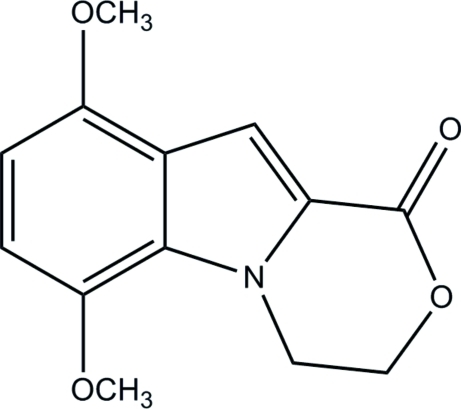


## Experimental

### 

#### Crystal data

C_13_H_13_NO_4_
*M* *_r_* = 247.24
Monoclinic, 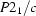

*a* = 8.414 (2) Å
*b* = 6.9722 (19) Å
*c* = 19.331 (5) Å
β = 101.276 (4)°
*V* = 1112.1 (5) Å^3^
*Z* = 4
Mo *K*α radiation
μ = 0.11 mm^−1^
*T* = 100 K
0.72 × 0.27 × 0.26 mm

#### Data collection

Bruker APEXII CCD diffractometer
Absorption correction: multi-scan (*SADABS*; Bruker, 2001 ▶) *T*_min_ = 0.925, *T*_max_ = 0.972
10088 measured reflections
2277 independent reflections
1922 reflections with *I* > 2σ(*I*)
*R*_int_ = 0.030

#### Refinement

*R*[*F* ^2^ > 2σ(*F* ^2^)] = 0.038
*wR*(*F* ^2^) = 0.102
*S* = 1.06
2277 reflections
165 parameters
H-atom parameters constrained
Δρ_max_ = 0.32 e Å^−3^
Δρ_min_ = −0.23 e Å^−3^

### 

Data collection: *APEX2* (Bruker, 2007 ▶); cell refinement: *SAINT* (Bruker, 2007 ▶); data reduction: *SAINT*; program(s) used to solve structure: *SIR97* (Altomare *et al.*, 1999 ▶); program(s) used to refine structure: *SHELXL97* (Sheldrick, 2008 ▶); molecular graphics: *ORTEP-3 for Windows* (Farrugia, 1997 ▶); software used to prepare material for publication: *WinGX* (Farrugia, 1999 ▶).

## Supplementary Material

Crystal structure: contains datablocks I, global. DOI: 10.1107/S1600536811000249/sj5085sup1.cif

Structure factors: contains datablocks I. DOI: 10.1107/S1600536811000249/sj5085Isup2.hkl

Additional supplementary materials:  crystallographic information; 3D view; checkCIF report

## Figures and Tables

**Table 1:** Hydrogen-bond geometry (Å, °)

*D*—H⋯*A*	*D*—H	H⋯*A*	*D*⋯*A*	*D*—H⋯*A*
C2—H2a⋯O16	0.99	2.40	2.9776 (18)	117
C3—H3*B*⋯O14^i^	0.99	2.56	3.2524 (19)	127 (4)
C15—H15*B*⋯O5^ii^	0.98	2.56	3.493 (2)	159 (4)
C17—H17*A*⋯O5^iii^	0.98	2.59	3.484 (2)	151 (4)

Symmetry codes: (i) 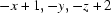; (ii) 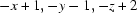; (iii) 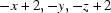.

**Fig. 1.**
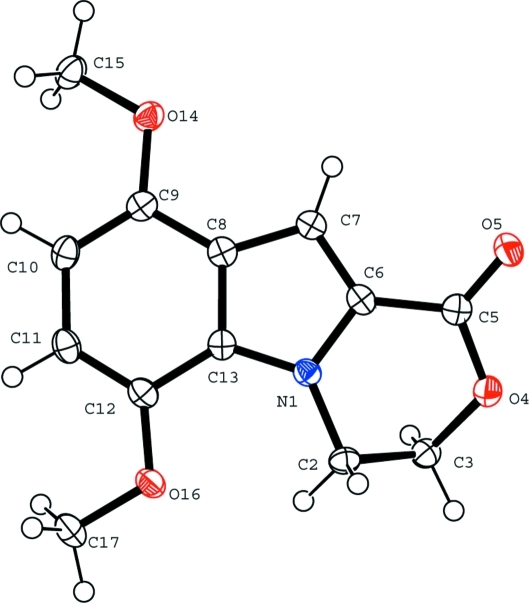
The structure of the title compound, with displacement ellipsoids drawn at the 50% probability level and H atoms with arbitrary radius.

**Fig. 2.**
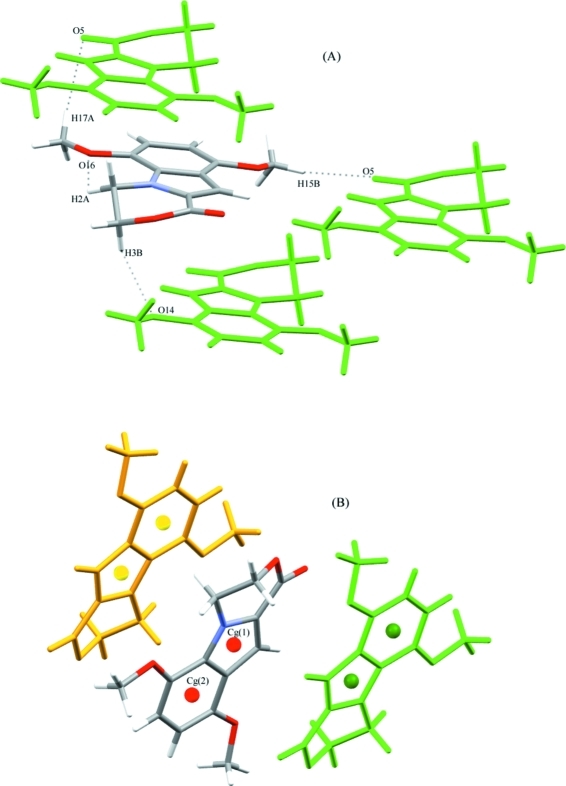
Intermolecular interactions in the crystal structure of the title compound, A) hydrogen-bonds, B) weak π-π interactions.

**Fig. 3.**
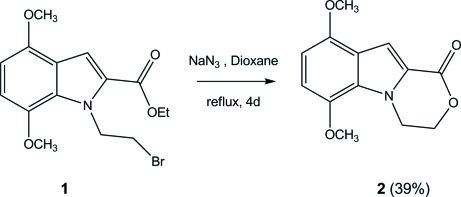
Reaction scheme for the preparation of molecule 2.

## supplementary crystallographic information

### Comment

Oxazinoindoles are very important as precursors of a wide range of natural and synthetic products with relevant biological properties such as antidepressant activity (Demerson *et al.*, 1975), 5-HT4 Receptor Antagonist (Fedouloff *et al.*, 2001), antiproliferative activity (Shchekotikhin *et al.*, 2004). The oxazinoindolone 2 is the product of the cyclization of ethyl 1-(2-bromoethyl)-4,7-dimethoxy-1*H*-indole-2-carboxylate mediated by the azido intermediate in dioxane at reflux (Fig. 2). Other efficient cyclizations have been reported also (Abbiati *et al.*,2005; Brudeli *et al.*, 2010; Fu *et al.*, 2010). The molecular structure of the title compound is represented in Fig. 1. Bond lengths and angles are within the expected values and confirm the bond orders giving in the Scheme. The e.s.d. for the molecular plane, as well as the bond distances and angles for the indol fragment, are within the expected values for bicyclic aromatic systems [r.m.s deviation = 0.006 (1) Å]. The shortest contacts between molecules are set along the crystallographic axis a, where the stacked molecules related by an inversion center form an ABAB array. Centroid to centroid distances range from 3.922 (2) to 4.396 (2)Å (Table 2). Weak C–H···O hydrogen bonds further stabilize the structure (Table 1).

### Experimental

6,9-Dimethoxy-3,4-dihydro-1*H*-[1,4]oxazino[4,3-*a*]indol-1-one (2)

Sodium azide (40 mg, 0.62 mmol) was added to a solution of ethyl 1-(2-bromoethyl)-4,7-dimethoxy-1*H*-indole-2-carboxylate 1 (100 mg, 0.28 mmol) in dioxane (5.0 ml) and the mixture was stirred at reflux for 4 days. The suspension was filtered and the solvent was removed *in vacuum* to give a residue, which was purified by flash column chromatography (CH_2_Cl_2_) to give 6,9-dimethoxy-3,4-dihydro-1*H*-[1,4]oxazino[4,3-*a*]indol-1-one (2) (27 mg, 39%) as a white solid. mp: 419.0–419.5 K (Fig. 3).

### Refinement

H atoms were placed in idealized positions with C—H distances 0.95 – 0.98 Å and thereafter treated as riding. A torsional parameter was refined for each methyl group. *U*iso for H were assigned as 1.2 times *U*eq of the attached C atom (1.5 for the methyl groups).

### Crystal data

**Table:**

C_13_H_13_NO_4_	*D*_x_ = 1.477 Mg m^−^^3^
*M**_r_* = 247.24	Melting point = 419.0–419.5 K
Monoclinic, *P*2_1_/*c*	Mo *K*α radiation, λ = 0.71073 Å
*a* = 8.414 (2) Å	Cell parameters from 1832 reflections
*b* = 6.9722 (19) Å	θ = 2.5–27.5°
*c* = 19.331 (5) Å	µ = 0.11 mm^−^^1^
β = 101.276 (4)°	*T* = 100 K
*V* = 1112.1 (5) Å^3^	Prism, colourless
*Z* = 4	0.72 × 0.27 × 0.26 mm
*F*(000) = 520	

### Data collection

**Table:**

Bruker APEXII CCD diffractometer	2277 independent reflections
Radiation source: fine-focus sealed tube	1922 reflections with *I* > 2σ(*I*)
graphite	*R*_int_ = 0.030
φ and ω scans	θ_max_ = 26.4°, θ_min_ = 2.2°
Absorption correction: multi-scan (*SADABS*; Bruker, 2001)	*h* = −10→10
*T*_min_ = 0.925, *T*_max_ = 0.972	*k* = 0→8
10088 measured reflections	*l* = 0→24

### Refinement

**Table:**

Refinement on *F*^2^	Primary atom site location: structure-invariant direct methods
Least-squares matrix: full	Secondary atom site location: difference Fourier map
*R*[*F*^2^ > 2σ(*F*^2^)] = 0.038	Hydrogen site location: inferred from neighbouring sites
*wR*(*F*^2^) = 0.102	H-atom parameters constrained
*S* = 1.06	*w* = 1/[σ^2^(*F*_o_^2^) + (0.0537*P*)^2^ + 0.3721*P*] where *P* = (*F*_o_^2^ + 2*F*_c_^2^)/3
2277 reflections	(Δ/σ)_max_ = 0.001
165 parameters	Δρ_max_ = 0.32 e Å^−^^3^
0 restraints	Δρ_min_ = −0.23 e Å^−^^3^

### Special details

**Table:**

Experimental. 6,9-Dimethoxy-3,4-dihydro-1*H*-[1,4]oxazino[4,3-*a*]indol-1-one (2) IR (NaCl, cm^-1^): 1730 (CO). ^1^H RMN (CDCl_3_, 200 MHz) d 3.89 (s, 6H, 3xOCH_3_); 4.63–4.76 (m, 4H, 2xCH_2_); 6.34 (d, 1H, *J* =8.3 Hz, H-6); 6.61 (d, 1H, *J* =8.3 Hz, H7); 7.50 (s, 1H, H9). ^13^C RMN (CDCl_3_, 50 MHz) d 42.8 (CH_2_); 55.6 (OCH_3_); 55.7 (OCH_3_); 66.9 (CH_2_); 99.1 (C9); 105.5 (C6); 108.5 (C7); 120.3 (C8a); 123.0 (C9a); 128.1 (C5a); 142.1 (C5); 148.7 (C8); 159.7 (CO). MS (CI) *m*/*z* 248.1 [(*M*+1)^+^, 100].
Geometry. All e.s.d.'s (except the e.s.d. in the dihedral angle between two l.s. planes) are estimated using the full covariance matrix. The cell e.s.d.'s are taken into account individually in the estimation of e.s.d.'s in distances, angles and torsion angles; correlations between e.s.d.'s in cell parameters are only used when they are defined by crystal symmetry. An approximate (isotropic) treatment of cell e.s.d.'s is used for estimating e.s.d.'s involving l.s. planes.
Refinement. Refinement of *F*^2^ against ALL reflections. The weighted *R*-factor *wR* and goodness of fit *S* are based on *F*^2^, conventional *R*-factors *R* are based on *F*, with *F* set to zero for negative *F*^2^. The threshold expression of *F*^2^ > σ(*F*^2^) is used only for calculating *R*-factors(gt) *etc*. and is not relevant to the choice of reflections for refinement. *R*-factors based on *F*^2^ are statistically about twice as large as those based on *F*, and *R*- factors based on ALL data will be even larger.

### Fractional atomic coordinates and isotropic or equivalent isotropic displacement parameters (Å^2^)

**Table:**

	*x*	*y*	*z*	*U*_iso_*/*U*_eq_	
N1	0.79680 (13)	0.12018 (17)	0.99864 (6)	0.0178 (3)	
C2	0.86881 (15)	0.3060 (2)	1.02003 (7)	0.0199 (3)	
H2A	0.8631	0.3907	0.9785	0.024*	
H2B	0.9839	0.2908	1.0432	0.024*	
C3	0.77300 (16)	0.3898 (2)	1.07063 (7)	0.0212 (3)	
H3A	0.8196	0.5157	1.0874	0.025*	
H3B	0.6598	0.4110	1.0458	0.025*	
O4	0.77361 (12)	0.26469 (14)	1.13077 (5)	0.0230 (2)	
O5	0.69846 (12)	−0.01995 (15)	1.16341 (5)	0.0258 (3)	
C5	0.72921 (15)	0.0798 (2)	1.11666 (7)	0.0197 (3)	
C6	0.72754 (15)	0.0118 (2)	1.04487 (7)	0.0183 (3)	
C7	0.66807 (15)	−0.1555 (2)	1.01289 (7)	0.0182 (3)	
H7	0.6138	−0.2542	1.0330	0.022*	
C8	0.70291 (15)	−0.1534 (2)	0.94399 (7)	0.0179 (3)	
C9	0.67102 (15)	−0.2832 (2)	0.88665 (7)	0.0192 (3)	
C10	0.72240 (16)	−0.2367 (2)	0.82573 (7)	0.0221 (3)	
H10	0.7025	−0.3227	0.7869	0.026*	
C11	0.80488 (16)	−0.0619 (2)	0.81999 (8)	0.0225 (3)	
H11	0.8394	−0.0340	0.7771	0.027*	
C12	0.83662 (15)	0.0683 (2)	0.87398 (7)	0.0195 (3)	
C13	0.78441 (15)	0.0205 (2)	0.93686 (7)	0.0176 (3)	
O14	0.59004 (11)	−0.44721 (14)	0.89811 (5)	0.0225 (2)	
C15	0.52860 (17)	−0.5606 (2)	0.83664 (8)	0.0259 (3)	
H15A	0.4598	−0.4806	0.8012	0.039*	
H15B	0.4647	−0.6675	0.8496	0.039*	
H15C	0.6194	−0.6109	0.8172	0.039*	
O16	0.91115 (11)	0.24306 (15)	0.87236 (5)	0.0228 (3)	
C17	0.94995 (18)	0.2967 (2)	0.80615 (7)	0.0256 (3)	
H17A	1.0370	0.2142	0.7961	0.038*	
H17B	0.9856	0.4307	0.8083	0.038*	
H17C	0.8538	0.2819	0.7687	0.038*	

### Atomic displacement parameters (Å^2^)

**Table:**

	*U*^11^	*U*^22^	*U*^33^	*U*^12^	*U*^13^	*U*^23^
N1	0.0153 (5)	0.0198 (6)	0.0184 (6)	−0.0006 (4)	0.0035 (4)	0.0009 (4)
C2	0.0163 (6)	0.0198 (7)	0.0227 (7)	−0.0024 (5)	0.0019 (5)	0.0000 (6)
C3	0.0218 (7)	0.0199 (7)	0.0212 (7)	0.0007 (5)	0.0023 (5)	0.0006 (6)
O4	0.0275 (5)	0.0220 (5)	0.0192 (5)	−0.0008 (4)	0.0039 (4)	−0.0007 (4)
O5	0.0297 (5)	0.0293 (6)	0.0185 (5)	−0.0027 (4)	0.0049 (4)	0.0024 (4)
C5	0.0145 (6)	0.0228 (7)	0.0208 (7)	0.0019 (5)	0.0010 (5)	0.0003 (6)
C6	0.0143 (6)	0.0220 (7)	0.0185 (7)	0.0032 (5)	0.0031 (5)	0.0034 (5)
C7	0.0137 (6)	0.0200 (7)	0.0204 (7)	0.0020 (5)	0.0023 (5)	0.0024 (5)
C8	0.0115 (6)	0.0215 (7)	0.0201 (7)	0.0035 (5)	0.0013 (5)	0.0014 (5)
C9	0.0127 (6)	0.0209 (7)	0.0235 (7)	0.0018 (5)	0.0020 (5)	−0.0010 (6)
C10	0.0175 (7)	0.0271 (8)	0.0214 (7)	0.0021 (6)	0.0032 (5)	−0.0051 (6)
C11	0.0180 (6)	0.0299 (8)	0.0206 (7)	0.0029 (6)	0.0062 (5)	0.0010 (6)
C12	0.0134 (6)	0.0241 (7)	0.0213 (7)	0.0013 (5)	0.0040 (5)	0.0024 (6)
C13	0.0127 (6)	0.0214 (7)	0.0180 (7)	0.0028 (5)	0.0013 (5)	0.0002 (5)
O14	0.0210 (5)	0.0230 (5)	0.0232 (5)	−0.0037 (4)	0.0040 (4)	−0.0039 (4)
C15	0.0229 (7)	0.0273 (8)	0.0273 (8)	−0.0038 (6)	0.0044 (6)	−0.0083 (6)
O16	0.0222 (5)	0.0270 (6)	0.0200 (5)	−0.0043 (4)	0.0063 (4)	0.0019 (4)
C17	0.0237 (7)	0.0331 (9)	0.0212 (7)	−0.0012 (6)	0.0076 (6)	0.0053 (6)

### Geometric parameters (Å, °)

**Table:**

N1—C13	1.3678 (17)	C9—C10	1.370 (2)
N1—C6	1.3830 (17)	C9—O14	1.3712 (17)
N1—C2	1.4557 (18)	C10—C11	1.418 (2)
C2—C3	1.5019 (19)	C10—H10	0.9500
C2—H2A	0.9900	C11—C12	1.369 (2)
C2—H2B	0.9900	C11—H11	0.9500
C3—O4	1.4525 (17)	C12—O16	1.3736 (18)
C3—H3A	0.9900	C12—C13	1.4108 (19)
C3—H3B	0.9900	O14—C15	1.4365 (17)
O4—C5	1.3552 (18)	C15—H15A	0.9800
O5—C5	1.2077 (17)	C15—H15B	0.9800
C5—C6	1.4638 (19)	C15—H15C	0.9800
C6—C7	1.368 (2)	O16—C17	1.4310 (17)
C7—C8	1.4188 (19)	C17—H17A	0.9800
C7—H7	0.9500	C17—H17B	0.9800
C8—C13	1.413 (2)	C17—H17C	0.9800
C8—C9	1.4156 (19)		
			
C13—N1—C6	108.48 (12)	C10—C9—C8	118.59 (13)
C13—N1—C2	131.04 (12)	O14—C9—C8	115.52 (12)
C6—N1—C2	120.48 (12)	C9—C10—C11	120.80 (13)
N1—C2—C3	106.52 (11)	C9—C10—H10	119.6
N1—C2—H2A	110.4	C11—C10—H10	119.6
C3—C2—H2A	110.4	C12—C11—C10	122.39 (13)
N1—C2—H2B	110.4	C12—C11—H11	118.8
C3—C2—H2B	110.4	C10—C11—H11	118.8
H2A—C2—H2B	108.6	C11—C12—O16	126.33 (13)
O4—C3—C2	111.70 (11)	C11—C12—C13	116.95 (13)
O4—C3—H3A	109.3	O16—C12—C13	116.70 (12)
C2—C3—H3A	109.3	N1—C13—C12	130.47 (13)
O4—C3—H3B	109.3	N1—C13—C8	107.84 (12)
C2—C3—H3B	109.3	C12—C13—C8	121.69 (12)
H3A—C3—H3B	107.9	C9—O14—C15	115.79 (11)
C5—O4—C3	116.89 (11)	O14—C15—H15A	109.5
O5—C5—O4	119.21 (13)	O14—C15—H15B	109.5
O5—C5—C6	124.02 (14)	H15A—C15—H15B	109.5
O4—C5—C6	116.73 (12)	O14—C15—H15C	109.5
C7—C6—N1	109.68 (12)	H15A—C15—H15C	109.5
C7—C6—C5	129.72 (13)	H15B—C15—H15C	109.5
N1—C6—C5	120.58 (13)	C12—O16—C17	115.93 (11)
C6—C7—C8	106.91 (12)	O16—C17—H17A	109.5
C6—C7—H7	126.5	O16—C17—H17B	109.5
C8—C7—H7	126.5	H17A—C17—H17B	109.5
C13—C8—C9	119.58 (13)	O16—C17—H17C	109.5
C13—C8—C7	107.08 (12)	H17A—C17—H17C	109.5
C9—C8—C7	133.33 (13)	H17B—C17—H17C	109.5
C10—C9—O14	125.89 (13)		
			
C13—N1—C2—C3	149.07 (13)	O14—C9—C10—C11	179.79 (12)
C6—N1—C2—C3	−32.03 (15)	C8—C9—C10—C11	−0.4 (2)
N1—C2—C3—O4	57.70 (13)	C9—C10—C11—C12	−0.3 (2)
C2—C3—O4—C5	−52.35 (15)	C10—C11—C12—O16	−177.91 (12)
C3—O4—C5—O5	−166.24 (12)	C10—C11—C12—C13	0.5 (2)
C3—O4—C5—C6	15.92 (16)	C6—N1—C13—C12	−179.71 (13)
C13—N1—C6—C7	−1.05 (15)	C2—N1—C13—C12	−0.7 (2)
C2—N1—C6—C7	179.83 (11)	C6—N1—C13—C8	1.00 (14)
C13—N1—C6—C5	177.50 (11)	C2—N1—C13—C8	180.00 (12)
C2—N1—C6—C5	−1.62 (18)	C11—C12—C13—N1	−179.28 (13)
O5—C5—C6—C7	12.2 (2)	O16—C12—C13—N1	−0.7 (2)
O4—C5—C6—C7	−170.05 (13)	C11—C12—C13—C8	−0.07 (19)
O5—C5—C6—N1	−166.01 (12)	O16—C12—C13—C8	178.48 (11)
O4—C5—C6—N1	11.72 (18)	C9—C8—C13—N1	178.81 (11)
N1—C6—C7—C8	0.66 (14)	C7—C8—C13—N1	−0.59 (14)
C5—C6—C7—C8	−177.72 (12)	C9—C8—C13—C12	−0.56 (19)
C6—C7—C8—C13	−0.04 (14)	C7—C8—C13—C12	−179.96 (12)
C6—C7—C8—C9	−179.33 (13)	C10—C9—O14—C15	−12.25 (19)
C13—C8—C9—C10	0.77 (18)	C8—C9—O14—C15	167.92 (12)
C7—C8—C9—C10	179.98 (13)	C11—C12—O16—C17	3.90 (19)
C13—C8—C9—O14	−179.38 (11)	C13—C12—O16—C17	−174.50 (11)
C7—C8—C9—O14	−0.2 (2)		

### Hydrogen-bond geometry (Å, °)

**Table:**

*D*—H···*A*	*D*—H	H···*A*	*D*···*A*	*D*—H···*A*
C2—H2a···O16	0.99	2.40	2.9776 (18)	117
C3—H3B···O14^i^	0.99	2.56	3.2524 (19)	127 (4)
C15—H15B···O5^ii^	0.98	2.56	3.493 (2)	159 (4)
C17—H17A···O5^iii^	0.98	2.59	3.484 (2)	151 (4)

Symmetry codes:  (i) −*x*+1, −*y*, −*z*+2; (ii) −*x*+1, −*y*−1, −*z*+2; (iii) −*x*+2, −*y*, −*z*+2.

### Table 2 Weak π-π intermolecular interactions.

**Table:**

Cg_I_-Cg_J_^*^	Cg_I_-Cg_J_(Å)^**^	Alpha(°)^***^	Beta(°)^****^	CgI_Perp(Å)^*****^
Cg(1)-Cg(1)	4.1164 (14)_(a)_	0	34.50	-3.3925 (6)
Cg(1)-Cg(1)	4.3962 (14)_(b)_	0	35.72	3.5692 (6)
Cg(1)-Cg(3)	4.6434 (15)_(b)_	0.50 (7)	40.18	3.5476 (6)

*Centroid plane numbers **Distance between ring centroids of planar cycles I and J. ***Dihedral angle between stacking planes. ****Angle Cg_I_-->Cg_J_ and normal to plane I. *****Perpendicular distance of Cg_I_ on ring J.Symmetry relationships: (a)1-x,-y,2-z (b)2-x,-y,2-z
